# Current concepts in eosinophilic esophagitis

**DOI:** 10.1007/s40629-017-0037-8

**Published:** 2017-09-13

**Authors:** Dagmar Simon, Alex Straumann, Alain M. Schoepfer, Hans-Uwe Simon

**Affiliations:** 1 Department of Dermatology, Bern University Hospital, Inselspital, University of Bern, 3010 Bern, Switzerland; 2 Swiss EoE Study Group, Olten, Switzerland; 3 Department of Gastroenterology and Hepatology, Lausanne University Hospital CHUV, Lausanne, Switzerland; 4 Institute of Pharmacology, University of Bern, 3010 Bern, Switzerland

**Keywords:** Eosinophilic esophagitis, Eosinophils, T‑helper 2 inflammation, Barrier dysfunction, Diet

## Abstract

**Background:**

Eosinophilic esophagitis (EoE) is a disease entity first described in the 1990s, but showing an increasing incidence that is characterized clinically by esophageal dysfunction and histologically by a striking eosinophil infiltration.

**Methods:**

This article discusses new aspects of the pathogenesis, symptoms, diagnosis, and treatment of EoE.

**Results:**

EoE affects both children and adults and is frequently associated with atopic disease and IgE sensitization. Barrier dysfunction and T‑helper 2 inflammation are considered to be pathogenetically important factors. Recently, a proton pump inhibitor (PPI)-sensitive EoE subtype as well as an EoE-like disorder have been described.

**Conclusion:**

Research in recent years has contributed to a better understanding of the disease spectrum and pathogenesis of EoE, including genetic dispositions, thereby laying the foundation for innovative treatment approaches.

## Introduction

In the early 1990s, several case series of adult and pediatric patients with dysphagia accompanied by eosinophil-rich inflammation on histology were described. They were classified as primary or idiopathic eosinophilic esophagitis (EoE) and differed from gastroesophageal reflux disease (GERD) [1, 2]. Our knowledge of EoE has grown continuously since then, and it is now recognized that we are dealing with a spectrum of diseases with a complex pathogenesis. This overview article is intended to discuss the various clinical and pathogenetic aspects of, and treatment options for, EoE.

## Definition of eosinophilic esophagitis

According to the updated guidelines, EoE is defined as a chronic, immune-/antigen-mediated disease limited clinically and pathologically to the esophagus. It is characterized by symptoms caused by esophageal dysfunction and, histologically, by eosinophilic inflammation. As a general rule, >15 eosinophils per high-power field (HPF; corresponding to 400× microscope magnification) are diagnostic for EoE [3].

## Rising incidence of eosinophilic esophagitis

EoE was initially considered a rare disease. However, gastroenterologists observed a steady increase in cases of newly diagnosed EoE. This raised the question of whether this increase could be attributed to a higher level of awareness or actually a higher incidence. An epidemiological study showed that there was indeed a rising incidence [4, 5]. In the Western world, one now assumes an incidence of 4.4–7.4 per 100,000 inhabitants/year and a prevalence of 43 per 100,000 [5]. A study in the US revealed an equally high prevalence in children [6]. Interestingly, men are the more commonly affected (male:female ratio of 3:1) [7].

## Differing symptoms in adults and children

EoE patients generally present in good general health and are primarily symptom-free. However, symptoms manifest upon ingestion of solid foods. Symptoms are typically age-dependent (Table 1). Whereas infants exhibit fussing, feeding problems, abdominal pain, and failure to thrive, older children experience dysphagia, chest pain, and spontaneous bolus obstruction. In adults, symptoms are limited to dysphagia upon eating hard, dry foods and after eating too fast, as well as occasional retrosternal pain. A third of all untreated EoE patients experience long-lasting bolus obstruction. By employing certain adaptive strategies, such as eating slowly, chewing food for a long time, flushing food down with fluids, as well as avoiding bread and meat, patients are able to cope with their disease and are unaware of their dysphagia and its potential clinical significance.

**Table 1:** Symptoms and findings in children and adults with eosinophilic esophagitis

	Children	Adults
*Symptoms*	Abdominal pain	Dysphagia
	Acid reflux	Bolus impaction
	Cough	Retrosternal pain
	Dysphagia	
	Regurgitation	
	Vomiting	
	Nausea	
	Pharyngitis/sore throat	
	Loss of appetite	
	Refusal to eat	
	Sleep disorders	
*Laboratory findings*	Blood eosinophilia	
	Elevated total IgE	
	Specific IgE to foods (milk, egg, wheat, peanut, fish)	Specific IgE to aeroallergens and pollen-related food allergens

## Diagnostic methods

**Fig. 1:**
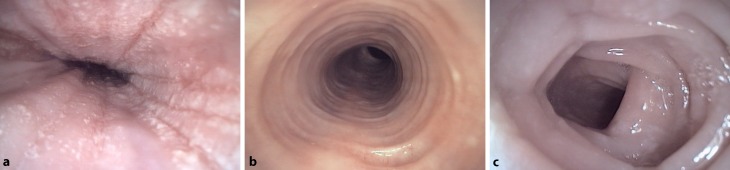
Endoscopic findings in eosinophilic esophagitis (EoE). **a** Acute inflammatory EoE with edema, white exudate, and furrows; **b** rings; **c** strictures in a chronic course

With the exception of occasional cases of failure to thrive in children, the clinical examination is generally normal, while peripheral blood eosinophilia may or may not be observed. Therefore, endoscopy and biopsies are essential for establishing a diagnosis [3]. A number of endoscopic findings are typical for EoE, yet non-pathognomonic: longitudinal furrows and exudates as a sign of acute inflammation, crêpe paper-like mucosa, as well as protruding ring-like structures and strictures in a chronic course (Table 2; Fig. 1; [9]). Endoscopy is combined with biopsy isolation. Since eosinophil inflammation does not affect the entire esophagus, but rather individual areas, it is important that biopsies be taken from a variety of proximal and distal sites. Taking at least six esophageal biopsies is recommended.

**Table 2:** Endoscopic and histological characteristics of eosinophilic esophagitis

	Finding	Interpretation
*Endoscopy*	Exudate	Inflammation
	Edema	Inflammation
	Longitudinal furrows/ridges	Inflammation
	Rings	Remodeling, fibrosis
	Strictures	Remodeling, fibrosis
*Histology*	Eosinophil infiltration (>15 eosinophils/HPF)	
	Eosinophil abscesses	
	Luminal eosinophil layer	
	Altered epithelial surface	
	Dilated intercellular spaces (spongiosis)	
	Dyskeratotic epithelial cells	
	Basal zone hyperplasia	
	Fibrosis of the lamina propria

Histology reveals a thickening of the epithelium and spongiosis (Table 2). Since the esophagus does not usually harbor eosinophils, their infiltration in the epithelium, where they are found as isolated cells, in groups, or even in small abscesses, is abnormal [9]. The same diagnostic threshold value of 15 eosinophils per HPF in areas of severe inflammation applies to both adults and children.

The complex and invasive procedure of endoscopy, isolation of biopsies, and histology is still indispensable for the diagnosis and follow-up of EoE, since reliable biomarkers or alternative, non-invasive test methods are not available in routine practice, though some patients can exhibit eosinophilia in peripheral blood.

In order to establish the severity of EoE, gastroenterologists use an endoscopy-based activity score [10]. This score shows relatively good concordance with a symptom-based patient score which uses an estimation of dysphagia, adaptive behavior due to EoE, and pain on swallowing [11]. However, in clinical remission, there may be a discrepancy between the symptom score and endoscopic and histological activity score [12].

## Impaired epithelial barrier and T‑helper 2 inflammation

Characterization of the inflammatory infiltrate and cytokine pattern showed that a T-helper 2 (Th2) reaction underlies EoE [13]. In addition to eosinophils, an increased number of interleukin (IL)-5 expressing T cells, B cells, mast cells with surface-bound IgE, and dendritic cells have been found in esophageal tissue. IL-5 increases the production, activation, and survival of eosinophils. In addition to tumor necrosis factor (TNF)-alpha, an increased expression of eotaxin, responsible for the recruitment of eosinophils in tissue, has been identified. Epithelial cells in EoE tissue produce increased levels of thymic stromal lymphopoietin (TSLP), which is known to trigger a Th2 immune response [14]. In line with this, it was shown that IL-13 plays an important role in the pathogenesis of EoE [15].

Impaired epithelial barrier function appears to be a key factor in EoE pathogenesis, similar to atopic dermatitis. An abnormal expression of desmogleins, claudin, cadherin, occludin, filaggrin, keratins, and antimicrobial peptides has been observed in EoE patients [14, 16–19]. Moreover, an imbalance of proteases, e. g., kallikrein 5 and 7, and protease inhibitors such as lymphoepithelial Kazal-type-related inhibitor (LEKTI), appears to be present (unpublished data). An inverse correlation between the expression levels of LEKTI and the number of eosinophils producing eosinophil extracellular traps (EETs) has been observed [14]. Using EETs, which are extracellular DNA traps that bind toxic granule proteins, eosinophils are able to kill bacteria in a targeted manner [20, 21]. Subepithelial deposits of extracellular matrix proteins and a subsequent remodeling and fibrosis are the main characteristics of EoE. It is likely that eosinophils are involved in this process, e. g., via the release of transforming growth factor (TGF)-beta [22]. The reduction in numbers of tissue eosinophils following treatment correlates with a reduction in fibrosis [23, 24].

**Fig. 2:**
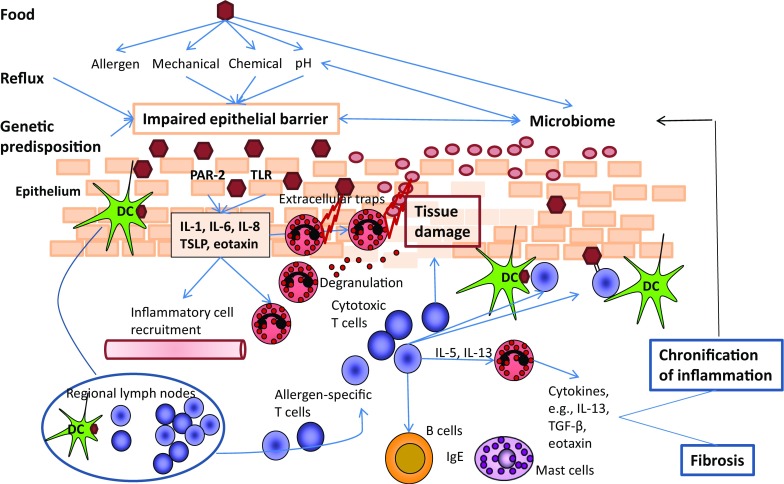
Pathomechanisms of eosinophilic esophagitis (EoE). Epithelial barrier impairment develops due to genetic predisposition and in consequence of reflux and food intake. Invading allergens and microbial antigens cause activation of the innate and acquired immune system. Eosinophils degranulate, release toxic proteins, and generate extracellular DNA traps, which serve as a defense system but also cause tissue damage. By releasing cytokines, eosinophils modulate inflammation and promote its chronification, ultimately with fibrosis, all of which, in turn, have negative effects on skin barrier function. *PAR-2* protease-activated receptor 2, *TLR* toll-like receptor, *DC* dendritic cell, *IL* interleukin, *TSLP* thymic stromal lymphopoietin, *TGF-β* transforming growth factor-beta, *IgE* immunoglobulin E

Thus, the following scenario seems reasonable (Fig. 2): owing to an impaired epithelial barrier, microbes and allergens are able to attach and or penetrate the epithelium. They are recognized by epithelial cells via pattern recognition receptors (PRR), which then release cytokines such as TSLP, which in turn initiate a Th2 inflammation. Eosinophils recruited to the tissue participate in defense by releasing toxic granule proteins via degranulation and/or in association with EETs. In addition, eosinophils stimulate the production of inappropriate extracellular matrix proteins and are thus involved in esophageal fibrosis in EoE. It is likely that eosinophils primarily perform a defense function by forming a second (toxic granule proteins) and, in the case of impaired epithelial barrier function, a third (fibrotic) barrier. Simultaneously, eosinophils can cause collateral damage to the esophageal epithelium through the release of the toxic granule proteins and contribute to the perpetuation of inflammation.

## Association with atopic disease

As early as the first publications describing EoE, mention was made of the frequent occurrence of concomitant atopic disease [2]. We have found such associations in 68% of adult EoE patients [25]. Interestingly, patients reported that their allergic airway disease manifested prior to EoE [25]. Studies with EoE in children and adults have shown that approximately 70% exhibit elevated total blood IgE levels [25, 26]. However, varying sensitization patterns depending on age have been observed. In children, sensitizations to food allergens such as milk, eggs, soy, wheat/rye, beef, and peanuts predominate [27]. The incidence of sensitization to foods declines with increasing age and sensitizations to pollen emerge [28]. IgE against environmental allergens are detected in around 90% of the adult EoE patients. Pollen allergens predominate among the sensitization patterns in adults, whereas sensitization to purely food allergens are rare [25]. In contrast, pollen-related food sensitizations—mostly to proteins homologous to the birch pollen allergens Bet v 1 and Bet v 2, namely PR-10 proteins and profilins—have been observed [29, 30]. It is worthy of note that a local immunoglobulin class switch and local IgE production can also occur in EoE [31].

## Genetics

The fact that EoE families exist, that male patients predominante, that most patients have atopic diseases and IgE sensitization, and that the Caucasian population is particularly affected suggests a genetic disposition. Interestingly, sporadic and familial EoE are extremely similar in terms of their clinical, endoscopic, and histopathological presentation, as well as their esophageal transcriptome profile [7]. The analysis of candidate genes revealed that single nucleotide polymorphisms (SNPs) of the eotaxin, TGF-beta, and filaggrin genes were striking in EoE patients [32–34]. Moreover, in addition to TSLP gene polymorphisms, a polymorphism in the TSLP receptor gene on Xp22.3 and Yp11.3 has been described, which would also at least partially explain EoE’s predominance in males [35]. A genome-wide association study identified the 5q22 and 2p23 loci spanning the gene for calpain 14, a protease expressed in the esophagus, as a risk factor for EoE [36, 37]. It can be assumed that EoE is a polygenetically determined disease with complex inheritance.

## EoE: a non-IgE-mediated food intolerance

The question is whether sensitizations, particularly those to food, play a role at all in the pathogenesis of EoE. Because of the high rate of sensitization and the clinical response to elimination diets, IgEs against food were suggested as playing a direct role. However, the determination of specific IgEs and/or skin prick tests appear inadequate to identify the EoE-eliciting allergens [27, 38]. The positive and negative predictive values of foods vary considerably, averaging 50–92% and 41–100%, respectively [39]. Diets geared towards eliminating these type I allergens do not by any means result in significant improvements of symptoms in all patients [27, 38–40]. Adult EoE patients sensitized to grass pollen, wheat, and rye did not respond to an elimination diet in which cereals were avoided over a 6-week period [40]. It should also be mentioned that administration of a monoclonal anti-IgE antibody did not result in a significant treatment effect in pediatric and adult EoE patients [41]. It is clear from clinical experimental data that EoE is not a typical, purely IgE-mediated disease; rather, one sees characteristics of EoE found in hypersensitivity reactions, in mostly T cell-mediated skin diseases, e. g., allergic contact dermatitis, drug eruptions, atopic dermatitis triggered by food, or chronic inflammatory bowel diseases [42]. In terms of clinical practice, this means that skin prick tests and the determination of specific IgE to food are not useful for identifying triggers of EoE. However, there is a rationale for their use to investigate concomitant allergic disease.

## New in the EoE spectrum: PPI-responsive esophageal eosinophilia

Like EoE, gastroesophageal reflux disease (GERD) can also be associated with eosinophil infiltration of the esophagus, which is why symptoms, treatment response, and histological response to PPI were formerly considered diagnostic criteria for GERD. A detailed investigation in patients with histologically proven eosinophil infiltration of the esophagus and response to PPI therapy showed that 75% achieved clinical remission, two thirds of whom exhibited a GERD profile (eosinophilic infiltration <35/HPF, signs of reflux on endoscopy, or pH measurement) and one third an EoE profile (eosinophilic infiltration >35/HPF, typical EoE symptoms, and endoscopy findings) [43]. At least a transient response to PPI has also been observed in children with EoE [44]. Transcriptome analysis was able to show that allergic inflammation resolves if esophageal eosinophilia responds to PPI [45]. The controversy over the extent to which PPI-responsive esophageal eosinophilia (PPI-REE) differs from EoE persists. According to a recently published consensus paper, the following arguments speak for PPI-REE more likely being a continuum of EoE rather than a separate entity [46]:

- There is no clinical, endoscopic, or histological distinction between PRI-REE and EoE.
- Th2-typical gene expression and inflammation are found in both PPI-REE and EoE patients.
- PPI reduces Th2 inflammation in PPI-REE in the same way as corticosteroids do with EoE.
- EoE patients may respond to diets and topical corticosteroids as well as to PPI.

## Recent discovery: an eosinophilic esophagitis-like disease

In recent years, we have observed members of four EoE families with an EoE-like disease [47]. They experienced symptoms including the esophageal dysfunction typical of EoE, but exhibited no, or only discrete, lesions in the esophageal mucosa on endoscopy. Histologically, T and mast cell infiltration was striking, while eosinophils were conspicuous for their absence. Less expression of eotaxin, TNF-alpha, and TSLP was noted as compared with EoE. Expression analysis of 94 mRNA transcripts showed a picture similar to EoE-like disease and EoE. However, mRNA expression of mucin 4 (*MUC4*) and cadherin 26 (*CDH26*) enabled a distinction between EoE-like disease and healthy controls, while another difference was seen between EoE and EoE-like disease in the mRNA expression of eotaxin. Interestingly, four of the five patients described with EoE-like disease were females, all of whom had offspring developing EoE in the first generation, suggesting the possibility of a hereditary disease. In summary, the evidence suggests that the two diseases have a similar pathogenesis.

## EoE in adults and children: one entity or distinct entities?

Epidemiological, clinical, and pathogenetic studies have always made a distinction between pediatric and adult EoE. Apart from clinical symptoms being more diffuse and the less likely occurrence of bolus obstruction in children, as well as the IgE sensitization spectrum, no significant differences are seen in terms of endoscopic findings, histology, pathogenesis, or response to drug therapy and dietary measures [8]. Remodeling resulting in the strictures and narrowing of the esophagus as seen by endoscopy is more frequently observed in adults as compared to children, which is most likely attributable to their longer duration of disease [48].

## Treatment

The treatment of EoE should have the following aims: remission of symptoms, control of inflammation, improvement of quality of life, prevention of hazardous bolus impaction, and, as a result, avoidance of long-term structural and functional damage (Table 3).

**Table 3:** Treatment of eosinophilic esophagitis

Intervention		Children	Adults	Comment
PPI		x	x	Initially 8–12 weeks
Corticosteroids	Systemic	x	x	–
	Oral	x	x	Initial and maintenance therapy
Diet	SFED	x	x	Re-exposure test
	FFED	x	x	Re-exposure test
	“Elemental”	x	x	–
Dilation		x	x	In stenosis, no anti-inflammatory effect
Bolus removal	Endoscopic	x	x	–

*SSFD* six-food elimination diet, *FFED* four-food elimination diet, *PPI *proton pump inhibitors

### Proton pump inhibitors

Since on average 60 and 50% of patients with esophageal eosinophilia respond to PPI treatment either with a clinical improvement or histological remission, respectively, PPI have recently been recommended for treatment initiation (1–2 standard doses once to twice daily for 8–12 weeks) [3, 49]. PPI are able to treat not only existing or concomitant GERD, but also PPI-REE. However, since only few studies on PPI are available as yet and there are also no reliable, direct comparative studies with the far better evaluated topical corticosteroids, it is not yet possible to position PPI in the treatment algorithm for EoE.

### Corticosteroids

A prospective controlled study in children showed that oral prednisone was as effective as high-dose topical fluticasone in achieving a clinical and histological remission of EoE [50]. Both topical and systemic corticosteroids are used for initial treatment in active EoE, as well as for maintenance treatment, whereby the type, strength, and dose are determined according to disease severity, availability, and practicability from the patient’s perspective [3]. Swallowed corticosteroids (budesonide, fluticasone) adhere well to the esophagus and are thus highly effective, as shown in studies with a treatment period of 2–12 weeks [51, 52]. Alternatively, corticosteroid aerosols can be used [53]. For adults, the results of a long-term treatment study (up to 50 weeks) with low-dose oral budesonide are available [24]. Inhibiting inflammation using corticosteroids reduces the risk of long-lasting bolus impactions [54].

### Biologicals

Based on the immunopathogenesis of EoE, several approaches for targeted treatment have emerged. The administration of mepolizumab, an anti-IL-5 antibody, achieved a marked reduction of blood and tissue eosinophils in both children and adults; the clinical effect, however, was minimal [23, 55]. Treatment with an anti-TNF-alpha antibody failed to show the expected effect, despite the fact that high TNF-alpha production by the epithelium is seen in EoE [56]. Although the majority of EoE patients exhibit IgE sensitization to direct or pollen-related food allergens, treatment with the anti-IgE antibody omalizumab did not elicit any significant effect in terms of reducing symptoms and inflammation, a finding which again supports the presumption that EoE is a non-IgE-mediated disease [41]. Thus, it is currently assumed that the pathogenesis of EoE is highly complex and that merely eliminating one mediator is not sufficient for an effective treatment. The results of IL-13 and/or IL-4 blockade are eagerly awaited. A pilot study on 23 patients with EoE demonstrated that treatment with an anti-IL-13 antibody was superior to placebo in terms of clinical and histological response [57].

### Diets

A study published as early as 1995 made the observation that children with EoE responded extremely well to an elemental diet comprised of amino acid-based dairy products; symptoms, however, recurred upon reintroduction of certain food proteins [58]. This elemental diet is highly effective, not only in EoE, but also in inflammatory bowel disease, which is why a generally anti-inflammatory mechanism is suspected [8]. Meanwhile, the effectiveness of an elemental diet has also been proven in adults [59]. Unfortunately, the practical implementation of an elemental diet is time-consuming and expensive. For this reason, it is used primarily in severe and treatment-refractory cases. In contrast, an empirical diet involving the elimination of milk, eggs, soy, wheat, peanuts, tree nuts, fish, and shellfish (six-food elimination diet) is able to resolve symptoms and histological changes in over 70% of children and adults affected by EoE [60, 61]. Re-exposure to wheat and milk (in 60 and 50% of cases, respectively) usually caused EoE recurrence, while concordance with specific IgE levels was seen in only 13% [61]. Recently, a four-food elimination diet, including the elimination of dairy products, wheat, eggs, and legumes, has been tested [62]. The clinicopathological remission rate was 54%; milk, eggs, and wheat were identified as triggers in 50, 36, and 31% of patients, respectively, in re-exposure testing [62].

### Dilation

Dilation is used for patients with an inadequate response to drug therapy or dietary measures, and in whom a functionally relevant narrowing of the esophagus has developed. When performed carefully using a flexible endoscope, dilation is a safe method [63]. It is important to note that dilation is a mechanical intervention and has no effect on eosinophilic inflammation.

## Tasks for the future

EoE has been known as a distinct disease entity for only around 25 years, and the effort that has gone into characterizing it clinically, endoscopically, and histologically, as well as into deciphering its pathogenesis and finding effective treatments, is enormous. What are the urgent questions/problems that need to be addressed?

- EoE can be considered as a spectrum of diseases characterized by eosinophil infiltration of the esophagus. What subtypes can be distinguished clinically, histologically, and in terms of treatment response? How can EoE be distinguished from other diseases, e. g., GERD, in the differential diagnosis?
- To date, endoscopy has been essential for establishing the diagnosis and monitoring treatment. There is an urgent need for non-invasive methods and biomarkers for routine practice. The development of the string test, in which inflammatory mediators that adhere to a piece of “swallowed” string are extracted and quantitatively measured, promises progress in this direction [64].
- What benefits can the use of omic technologies, e. g., creating transcriptome profiles for blood and tissue, confer?
- Changes in the esophageal microbiome, such as an absolute increase in the number of bacteria and a relative predominance of *Haemophilus *have been identified in EoE [65]. What are the effects of these changes and how can they be prevented or regulated?
- EoE is associated with food intolerance [42]. What are the underlying mechanisms? What are the diagnostic possibilities and dietary consequences?
- The effects of treatments used to date, e. g., corticosteroids, are broad and non-specific. Future treatments should interfere with the pathomechanism of EoE in a targeted manner, i. e., should be effective with few side effects and be practical in their application.

## Abbreviations

EETs: Eosinophil extracellular (DNA) trap
EoE: Eosinophilic esophagitis
GERD: Gastroesophageal reflux disease
HPF: High-power field
IgE: Immunoglobulin E
IL: Interleukin
LEKTI: Lymphoepithelial Kazal-type-related inhibitor
MRNA: Messenger ribonucleic acid
PRR: Pattern recognition receptor
PPI: Proton pump inhibitors
PPI-REE: Proton pump inhibitor-responsive esophageal eosinophilia
SNP: Single nucleotide polymorphism
TGF: Transforming growth factor
TNF: Tumor necrosis factor
TSLP: Thymic stromal lymphopoietin (cytokine)
